# The trajectory of carbon emissions and terrestrial carbon sinks at the provincial level in China

**DOI:** 10.1038/s41598-024-55868-y

**Published:** 2024-03-09

**Authors:** Yongjie Hu, Ying Li, Hong Zhang, Xiaolin Liu, Yixian Zheng, He Gong

**Affiliations:** 1 Sinopec International Petroleum Exploration and Production Corporation, Beijing, 100029 China; 2 School of Earth Sciences and Engineering, Nanjing University, Nanjing, 210093 Jiangsu China; 3 Merchant Marine College, Shanghai Maritime University, Shanghai, 201306 China; 4 Key Laboratory of Ecosystem Network Observation and Modeling, Institute of Geographic Sciences and Natural Resources Research, Chinese Academy of Sciences, Beijing, 100101 China

**Keywords:** Ecology, Environmental sciences, Environmental social sciences

## Abstract

Global greenhouse gas emission, major factor driving climate change, has been increasing since nineteenth century. STIRPAT and CEVSA models were performed to estimate the carbon emission peaks and terrestrial ecosystem carbon sinks at the provincial level in China, respectively. Utilizing the growth characteristics and the peak time criteria for the period 1997–2019, the patterns of energy consumption and CO_2_ emissions from 30 Chinese provinces are categorized into four groups: (i) one-stage increase (5 provinces), (ii) two-stage increase (10 provinces), (iii) maximum around 2013 (13 provinces), and (iv) maximum around 2017 (2 provinces). According to the STIRPAT model, the anticipated time of peak CO_2_ emissions for Beijing from the third group is ~ 2025 in both business-as-usual and high-speed scenarios. For Xinjiang Uygur autonomous region from the first group and Zhejiang province from the second group, the expected peak time is 2025 to 2030. Shaanxi province from the fourth group is likely to reach carbon emission peak before 2030. The inventory-based estimate of China’s terrestrial carbon sink is ~ 266.2 Tg C/a during the period 1982–2015, offsetting 18.3% of contemporary CO_2_ emissions. The province-level CO_2_ emissions, peak emissions and terrestrial carbon sinks estimates presented here are significant for those concerned with carbon neutrality.

## Introduction

Global greenhouse gas emissions have been increasing since the nineteenth century, recognized as a major factor driving global warming and climate change. More than 127 countries, especially parties to the Paris Agreement, have made individual climate pledges to cut down greenhouse gas emissions since 2015^1,2^. China pledges to achieve the peak of carbon dioxide (CO_2_) emissions before 2030, and carbon neutrality before 2060. This forces China to take the lead in reducing CO_2_ emissions to net zero in the long run^3^. Understanding the balance between Chinese carbon emission and sink is crucial to achieve net zero carbon emissions.

Energy consumption is the predominant source of greenhouse gas emissions. In China, coal accounts for ~ 56.8% of total energy consumption in 2020^4^. The high-emitting and low-efficiency utilization of coal, together with construction and cement production, positions China currently being the largest emitter of CO_2_ in the world^5^. Despite comprehensive summaries of China’s energy consumption and CO_2_ emissions growth patterns in the past decades^6–14^, discrepancies arise in gigatonnes gap of CO_2_ emissions due to different energy datasets. Notably, province-level growth patterns are limited reported. With respect to the future trajectories of China’s CO_2_ emissions, several approaches (e.g., environmental Kuznets Curves, grey model, Markov model, and STIRPAT model) were performed to predicate the optimal year to reach carbon emissions peak and cumulative emissions^15–17^. Significant differences of the optimal year and peak emissions are presented in these studies due to the different scenarios’ setup, calculation models and regression methods.

Terrestrial ecosystem (e.g., forests, agriculture, grasslands, and wetlands) is closely coupled to the climate system. This ecosystem is suggested to be the most cost-effective and readily available player to absorb CO_2_ and other greenhouse gases^18,19^. Numerous estimates and models have been employed to calculate China’s terrestrial carbon sinks, yet consensus remains elusive due to the uncertainty of parameters and observations^20–25^. These studies reveal that China’s terrestrial carbon sinks have a wide range from 70 to 1910 Tg C/a, and mostly fall into the range from 100 to 450 Tg C/a. Regional estimates of terrestrial carbon sinks have also been conducted, exposing significant disparities in different studies, such as in Qinghai-Tibet Plateau (43.16 Tg C/a^26^ vs. 182 Tg C/a^27^) and Guangdong province (53.2–54.5 Tg C/a^28^ vs. 69 Tg C/a^29^).

To estimate province-level carbon emissions peak and terrestrial carbon sinks, STIRPAT (Stochastic Impacts by Regression on Population, Affluence, and Technology) and CEVSA (Carbon Exchange between Vegetation, Soil, and the Atmosphere) models are performed in this study. China and provinces-level energy consumption for the period 1990–2020 and CO_2_ emissions values for the period 1997–2019 are also presented. While China comprises 34 provinces (autonomous regions and municipalities), this study focused on 30 provinces for calculating their CO_2_ emissions and 33 provinces for assessing terrestrial ecosystems carbon sinks, based on the currently available data. This study aims to: (i) present the growth patterns of energy consumption and CO_2_ emissions in 30 provinces; (ii) estimate carbon emissions peak and corresponding time for 4 typical provinces; and (iii) calculate 33 provinces’ terrestrial ecosystems carbon sinks and also discuss their implications for achieving carbon neutrality.

## Methods and data

### Energy-related carbon emissions accounting

Following previous methods^7,8^, CO_2_ emissions generated by energy consumption were calculated as follows:

1 $${\text{CO}}_{{2}} \;{\text{emission}} = \sum {\text{CS}}_{x} \times {\text{ NCV}} \times {\text{CC}} \times {\text{OE}} \times {44}/{12}$$

where CS refers to the fossil fuels consumption by the fuel type *x*; NCV represents net caloric value generated by per unit of consumption; CC refers to CO_2_ emission per net caloric value by fuel; and OE is the oxygenation efficiency, respectively. Moreover, the increment value and rate of provincial CO_2_ emissions were defined as follows:

2 $${\text{Increment}}\;{\text{value}} = {\text{ EMISSION}}_{ab} - {\text{ EMISSION}}_{ac}$$

3 $${\text{Increment}}\;{\text{rate}} = \left( {{\text{EMISSION}}_{ab} - {\text{EMISSION}}_{ac} } \right)/{\text{EMISSION}}_{ac} \times {1}00\%$$

where EMISSION represents CO_2_ emissions of a province in one year; the subscript *a* rerefers to provinces; and *b* as well as *c* is a specified year.

### STIRPAT model and scenarios

Compared to other methods (e.g., logarithmic mean Divisia index (LMDI)), STIRPAT model could examine more impact factors towards environment^30^. This model has become an increasingly dominant method in examining the impact factors for CO_2_ emissions^17^. This is a stochastic regression model related to population, affluence and technology parameters, defined as follows:

4 $${\text{I}} = {\text{aP}}^{{\text{b}}} {\text{A}}^{{\text{c}}} {\text{T}}^{{\text{d}}} {\text{e}}$$

where *I* is a given environmental indicator; *P* is population; *A* is affluence (i.e., GDP per capita); *T* is technology; *a* is a constant term; *b*, *c* and *d* are the exponential parameters estimated; and *e* is the random error, respectively. This formula (4) could be re-written as follows:

5 $${\text{ln}}I = {\text{ln}}a + b\left( {{\text{ln}}P} \right) + c\left( {{\text{ln}}A} \right) + d\left( {{\text{ln}}T} \right) + {\text{ln}}e$$

where *I* is replaced by *C* (carbon emissions) in this study. Following previous studies^17,31,32^ and integrating additional factors, STIRPAT model is extended as follows:

6 $${\text{ln}}C = {\text{ln}}a + b\left( {{\text{ln}}P} \right) + c\left( {{\text{ln}}A} \right) + d\left( {{\text{ln}}ET} \right) + f\left( {{\text{ln}}EC} \right) + g\left( {{\text{ln}}ES} \right) + h\left( {{\text{ln}}IS} \right) + i\left( {{\text{ln}}UR} \right) + {\text{ln}}e$$

where *ET* is carbon emission intensity; *EC* is energy consumption intensity (tonne of standard coal equivalent/ten thousand RMB); *ES* is energy structure (coal consumption/total energy consumption); *IS* is industrial structure (secondary industry/GDP); *UR* is urbanization rate; *f*, *g*, *h* and *i* are the exponential parameters estimated, respectively.

Due to the multicollinearity of above independent variables, the ordinary least square (OLS) regression generally fails to bring forward the reliable relationship between carbon dioxide emissions and those factors. Thus, ridge regression^33^ is used here to improve the accuracy and reliability of parameter estimation.

To better estimate provincial carbon emissions trajectories, three types of scenarios/ sensitivity analysis, including high-speed, business-as-usual (BAU) and low-speed, are performed here. In the BAU model, the annual increment rates of factors are set in accordance with the 14th Five-Year Plan related to energy transition, urbanisation, and investment^34,35^ and 2035 domestic goals. Compared to BAU scenario, increment rates are set somewhat lower or higher than those in low-speed and high-speed scenarios, respectively. The detailed increment rates of these factors of four provinces (i.e., Xinjiang, Zhejiang, Beijing, and Shaanxi provinces) are listed in supplementary Table S1.

### CEVSA model

Carbon fluxes in terrestrial ecosystem is controlled by eco-physiological and environmental factors, such as vegetation pattern and structure, photosynthesis, temperature, water, and nutrients. To estimate the controls of these factors on carbon fluxes, CEVSA model is used in this study. This model is predominantly composed of a biophysical sector calculating the transfers of heat and water; a plant growth sector related to photosynthesis, autotrophic respiration, carbon and nitrogen allocation and accumulation among plant organs, leaf area index and litter production; and a soil sector simulating decomposition of organic carbon and the inputs and outputs of nitrogen. Detailed descriptions, explanations, parametrization, and calculations for this model have been documented in previous work^18,36^. Using observation-based data of climate, vegetation, soil, and atmospheric CO_2_ for the period 1982 to 2015, the CEVSA model was run to analyse the terrestrial carbon sinks of 33 provinces.

### Data

Chinese energy consumption and CO_2_ emission data are derived from earlier studies^37–39^. Additionally, 30 provinces-level energy consumption and CO_2_ emission data are primarily obtained from previous studies^4,7,8,40^. No data in Tibet, Hong Kong, Macao, and Taiwan. Due to data availability, the research period for energy consumption and CO_2_ emission data are from 1990 to 2020 and from 1997 to 2019, respectively.

Population, GDP per capita, carbon emission intensity, energy consumption intensity, energy structure, industrial structure, and urbanization rate data of four provinces used in STIRPAT scenarios are from the 14th Five-Year Plan and 2035 domestic goals, available on provincial government websites (e.g., https://www.beijing.gov.cn/gongkai/shuju/) and previous studies^4,7,8^. Primary data performed in CEVSA model are collected from National Bureau of Statistics of China. The research period used in CEVSA model is from 1982 to 2015 due to data availability.

## Result

### ***Energy consumption and CO***_***2***_*** emissions***

#### National and provincial level for the past 23 years

**Figure 1:**
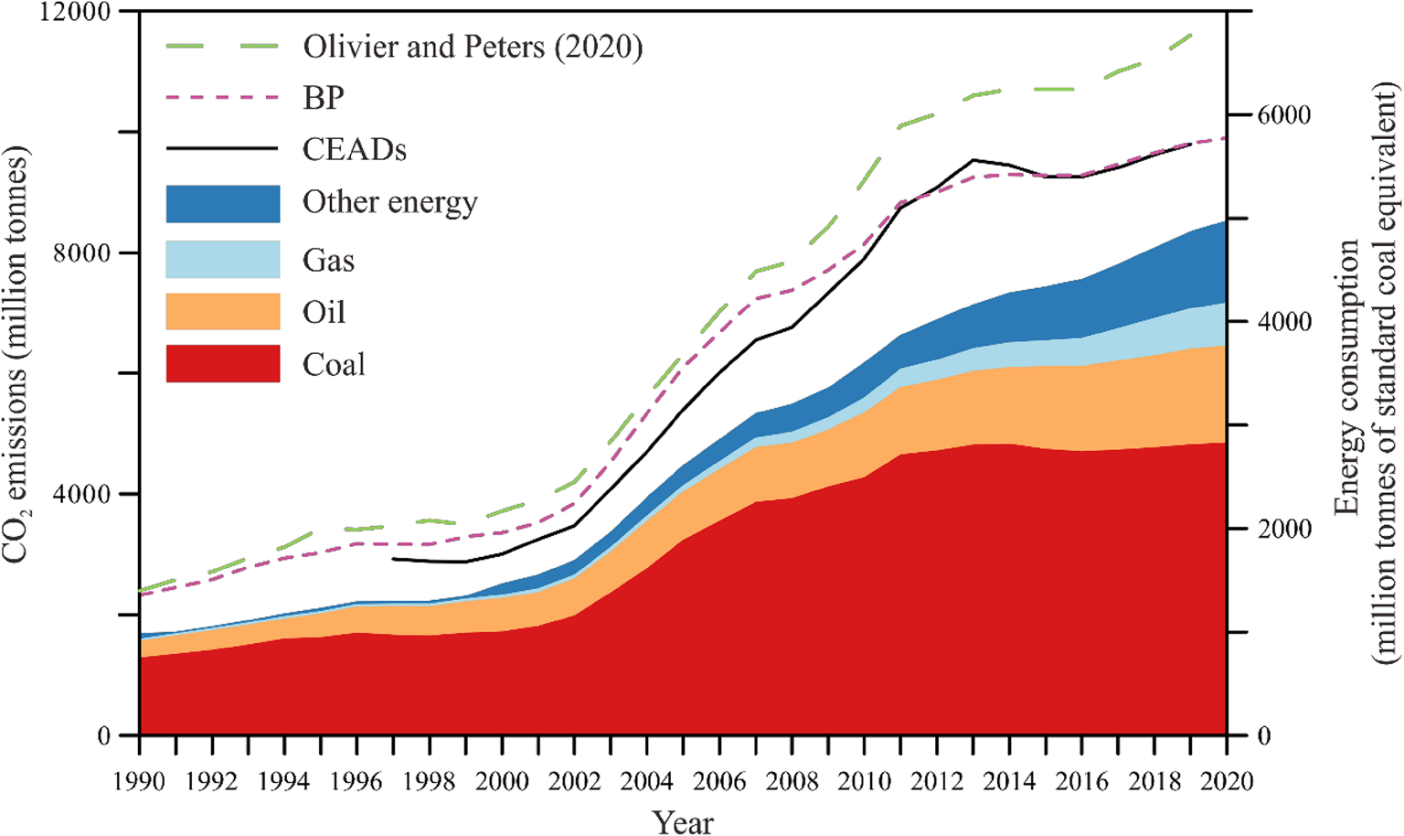
Energy consumption (million tonnes of standard coal equivalent) and CO_2_ emissions (million tonnes) of China for the period 1990–2020. Other energy includes hydroelectricity, renewables, and nuclear. Data are from previous studies^4,37–39^.

Energy consumption in China shows a slow increase from 1990 to 2002, a significant increase from 2003 to 2013, and a slight increase since 2013 (Fig. 1). Coal is the largest share in the total consumption, and its share yields a significant decrease from 72.5% in 2007 to a value of 56.8% in 2020. Meanwhile, the percentage of oil shows a stable increase from 16.4% in 2009 to 18.9% in 2020. Gas and other energy (hydroelectricity, renewables, and nuclear) have share values of 8.4% and 15.9%, respectively, in 2020.

**Figure 2:**
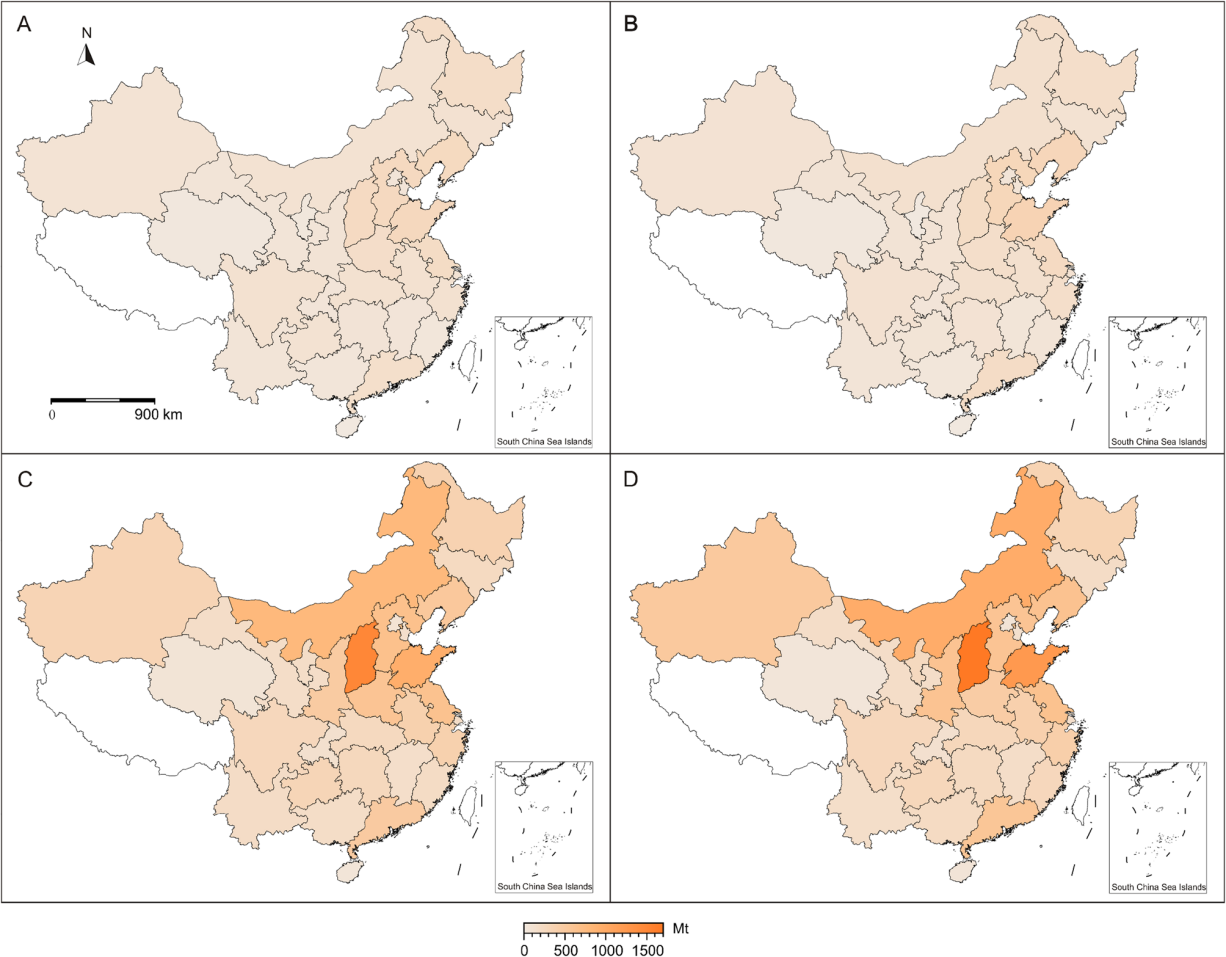
China province-level CO_2_ emissions in four years. (**A**) CO_2_ emissions in 1997. (**B**) CO_2_ emissions in 2002. (**C**) CO_2_ emissions in 2013. (**D**) CO_2_ emissions in 2019. No data in Tibet, Hong Kong, Macao, and Taiwan. The provinces name is referred to Fig. 5. All maps were drawn by the Generic Mapping Tools package (version 5.4.1) based on the standard map of China No. GS (2023) 2767.

Likewise, CO_2_ emissions in China is characterized by a slow growth from 1990 to 2002, a significant increase from 2002 to 2013, and a slight increase since 2013 (Fig. 1). Compared to 1997, 28 provinces yield similar CO_2_ emissions in 2002 (Fig. 2A,B). From 2002 to 2013, these provinces’ CO_2_ emissions show a robust increase with an average increment rate of 210% (Fig. 2C). In detail, Shanxi and Inner Mongolia yield the highest (620%) and second highest (490%) rate, respectively. 30 provinces (adding Ningxia and Hainan provinces) yield similar CO_2_ emissions in 2019 to their counterpart in 2013 (Fig. 2D).

**Figure 3:**
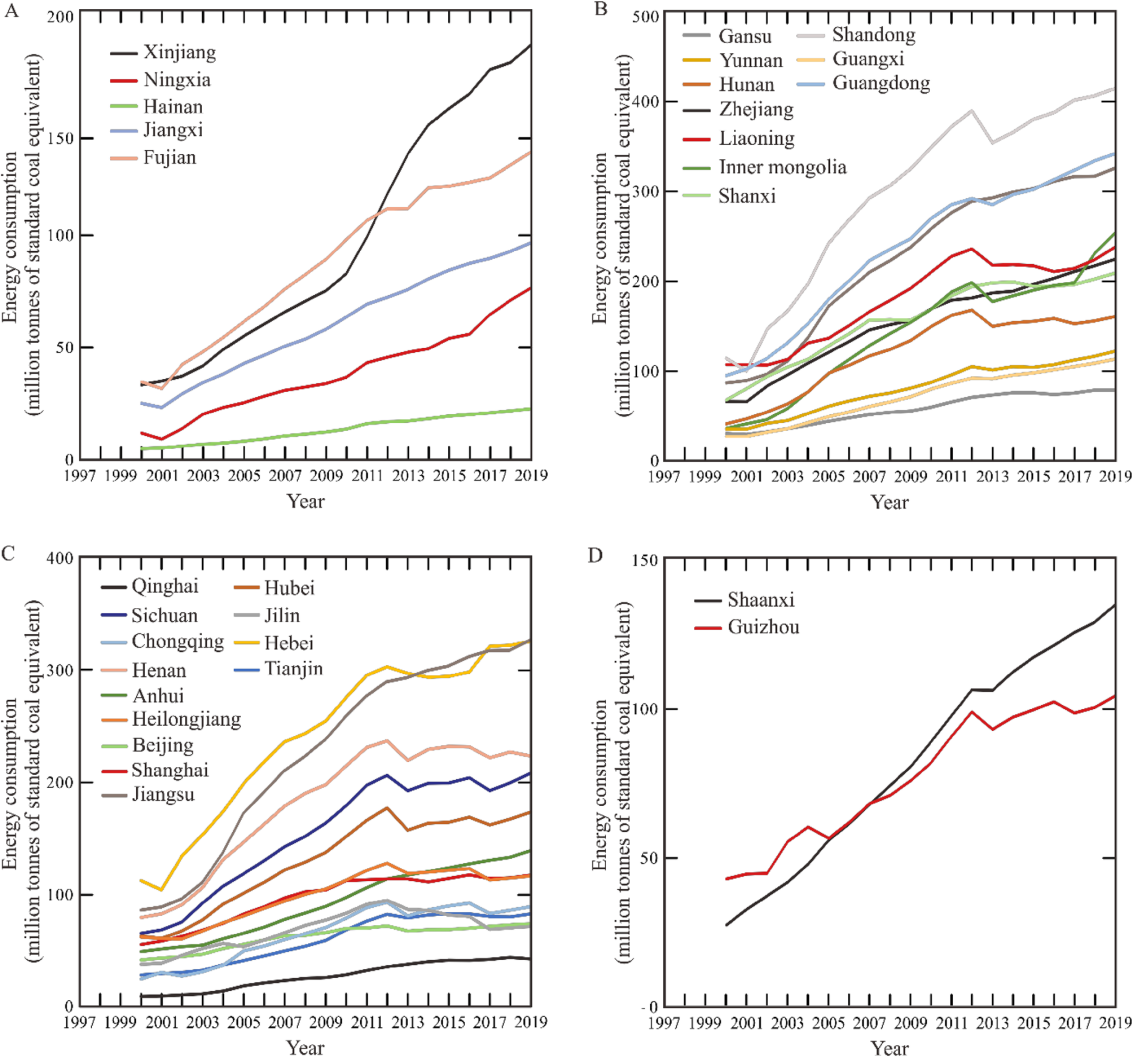
China provinces-level energy consumption (million tonnes of standard coal equivalent) for the period 2000–2019. (**A**) The energy consumption of five provinces from “one-stage increasing” group. (**B**) The energy consumption of ten provinces from “two-stage increasing” group. (**C**) The energy consumption of 13 provinces from “maximum around 2013” group. (**D**) The energy consumption of two provinces from “maximum around 2017” group.

**Figure 4:**
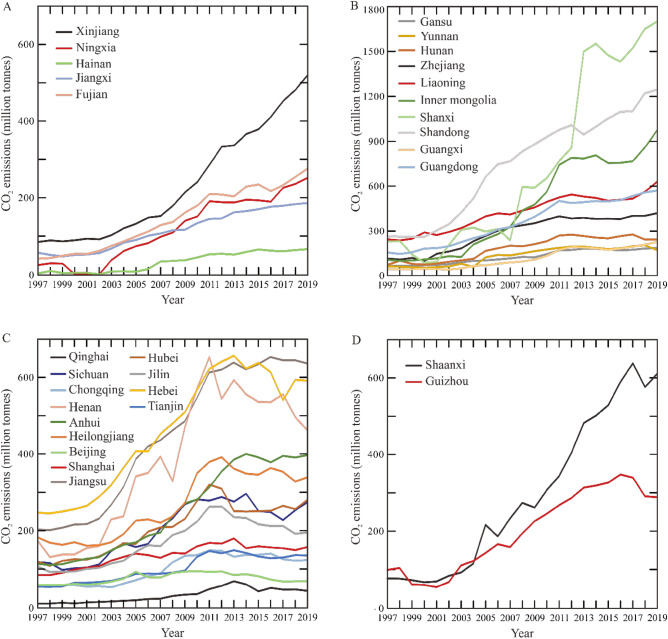
China provinces-level CO_2_ emissions (million tonnes) for the period 1997–2019. (**A**) CO_2_ emissions of five provinces from “one-stage increasing” group. (**B**) CO_2_ emissions of ten provinces from “two-stage increasing” group. (**C**) CO_2_ emissions of 13 provinces from “maximum around 2013” group. (**D**) CO_2_ emissions of two provinces from “maximum around 2017” group.

**Figure 5:**
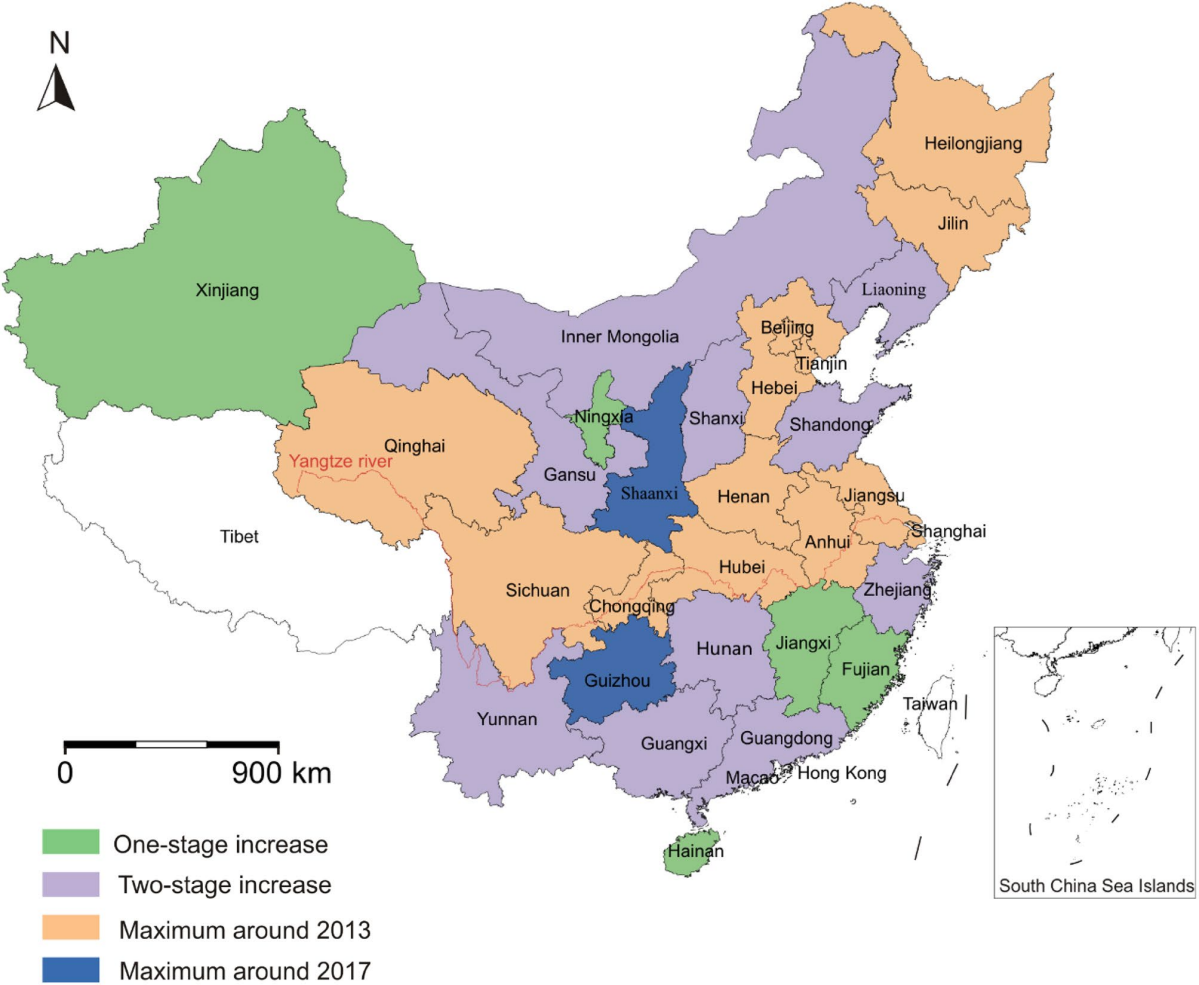
Distributions of four groups of provincial energy consumption and CO_2_ emissions in China. No data in Tibet, Hong Kong, Macao, and Taiwan. Map was drawn by the Generic Mapping Tools package (version 5.4.1) based on the standard map of China No. GS (2023) 2767.

Based on the growth variation and the time of reaching the maximum for the period 1997–2019, the growth patterns of energy consumption and CO_2_ emissions from 30 China provinces are divided into four groups (Figs. 3, 4): (i) one-stage increase; (ii) two-stage increase; (iii) maximum around 2013, and (iv) maximum around 2017. Five provinces (Xinjiang, Ningxia, Jiangxi, Fujian and Hainan) are characterized by “one-stage increase” of both energy consumption and CO_2_ emissions in the past 23 years. These provinces are predominantly located in the northwestern and southern China (Fig. 5). Xinjiang Uygur autonomous region is a typical example from the first group due to its stably increasing carbon emissions. 10 provinces (Gansu, Yunnan, Hunan, Zhejiang, Liaoning, Inner Mongolia, Shanxi, Shandong, Guangxi, and Guangdong) show features of two-stage increase and are generally located in the northern and southern China. “Two-stage increase” is that both the energy consumption and CO_2_ emissions generally show a stable increase from 2000 to 2012, and a slight increase from 2014 to 2020. Zhejiang province is characterized by the intermediate CO_2_ emissions among the 10 provinces.

The third group consists of 13 provinces, including Qinghai, Sichuan, Chongqing, Henan, Anhui, Heilongjiang, Beijing, Shanghai, Jiangsu, Hubei, Jilin, Hebei and Tianjin, dominantly located along Yangtze River or in the northern China. These provinces reach the peak of both historic energy consumption and CO_2_ emissions in 2012 or 2013. Beijing city yields stable energy consumption after reaching the maximum. The fourth group is composed of Shaanxi and Guizhou provinces, whose historic CO_2_ emissions reach the maximum in 2016 or 2017. Compared to Guizhou province, Shaanxi province is characterized by significantly high energy consumption and carbon emissions.

#### Peak carbon emissions and times of 4 provinces

**Figure 6:**
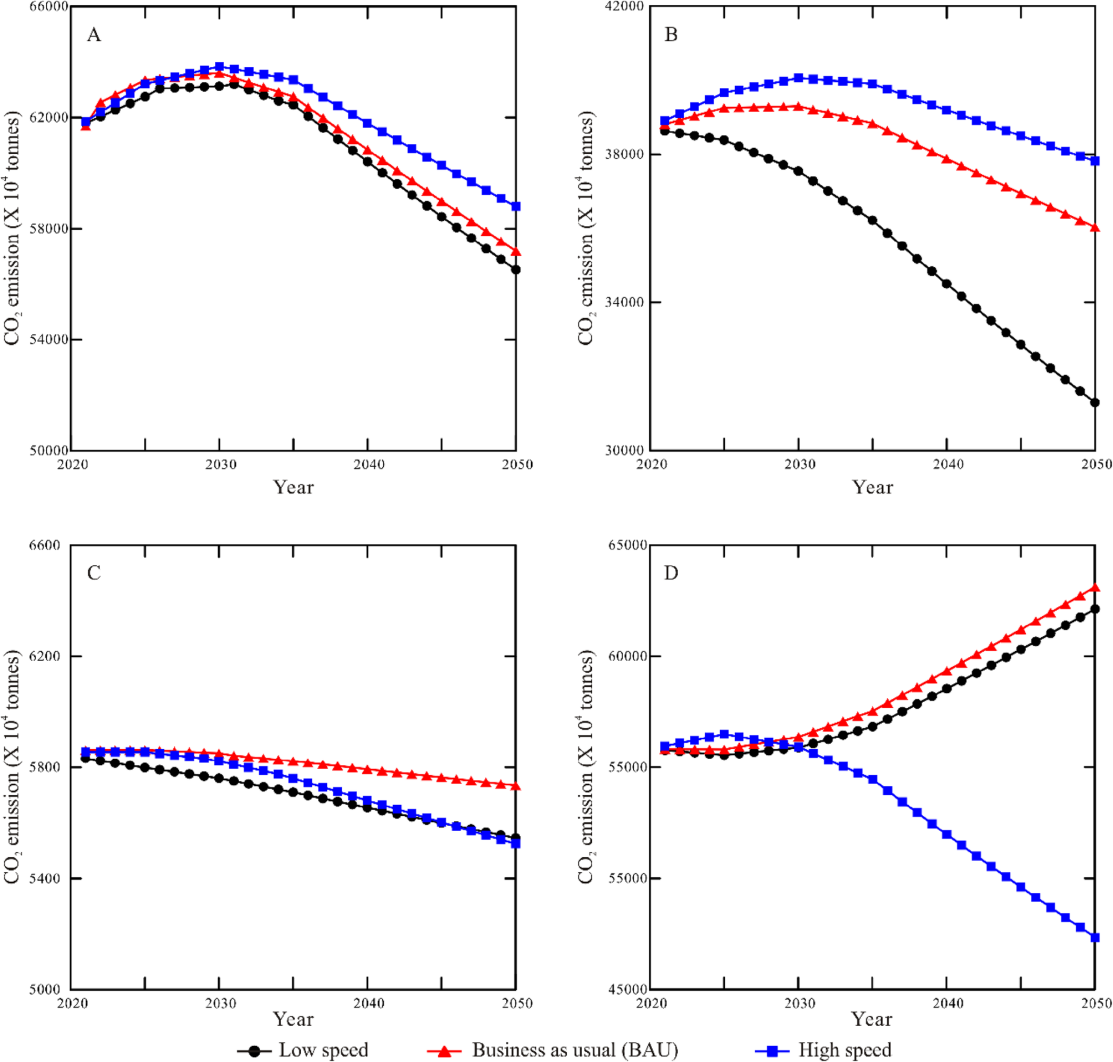
Energy-related carbon dioxide emissions of 4 regions and provinces in low speed, business-as-usual (BAU) and high speed scenarios for the period 2020–2050. (**A**) Energy-related carbon dioxide emissions of Xinjiang Uygur autonomous region in three different scenarios for the period 2020–2050. (**B**) Energy-related carbon dioxide emissions of Zhejiang province in three different scenarios for the period 2020–2050. (**C**) Energy-related carbon dioxide emissions of Beijing city in three different scenarios for the period 2020–2050. (**D**) Energy-related carbon dioxide emissions of Shaanxi province in three different scenarios for the period 2020–2050.

STIRPAT model shows that Xinjiang, an example of the “one-stage increase” group, might reach peak emission around 2030 in the context of three scenarios (Fig. 6A). Zhejiang province from the “two-stage increase” group is estimated to achieve peak emissions around 2025–2030 in three conditions (Fig. 6B). Beijing, the case study for the “maximum around 2013” group, likely reaches peak emission around 2020 in low-speed setting, and yields peak values around 2025 in the context of BAU and high-speed scenarios (Fig. 6C). The optimal year of peak emissions for Shaanxi province from the fourth group is around 2025 in high-speed scenario (Fig. 6D).

Prior to peak emissions, the cumulative CO_2_ emissions in high-speed scenario of these four provinces are higher than those in low-speed and BAU scenarios. This tendency is further shown in Xinjiang Uygur autonomous region and Zhejiang province after reaching the peak of emissions. However, Beijing and Shaanxi provinces have cumulative CO_2_ emissions of high-speed scenario lower than those in the other conditions after emissions peak. In the context of the three scenarios, the lowest peak CO_2_ emissions for Xinjiang, Zhejiang, Beijing, and Shaanxi are 636.06, 386.94, 58.37 and 564.90 Mt, respectively.

### ***Terrestrial ecosystems CO***_***2***_*** sinks***

**Figure 7:**
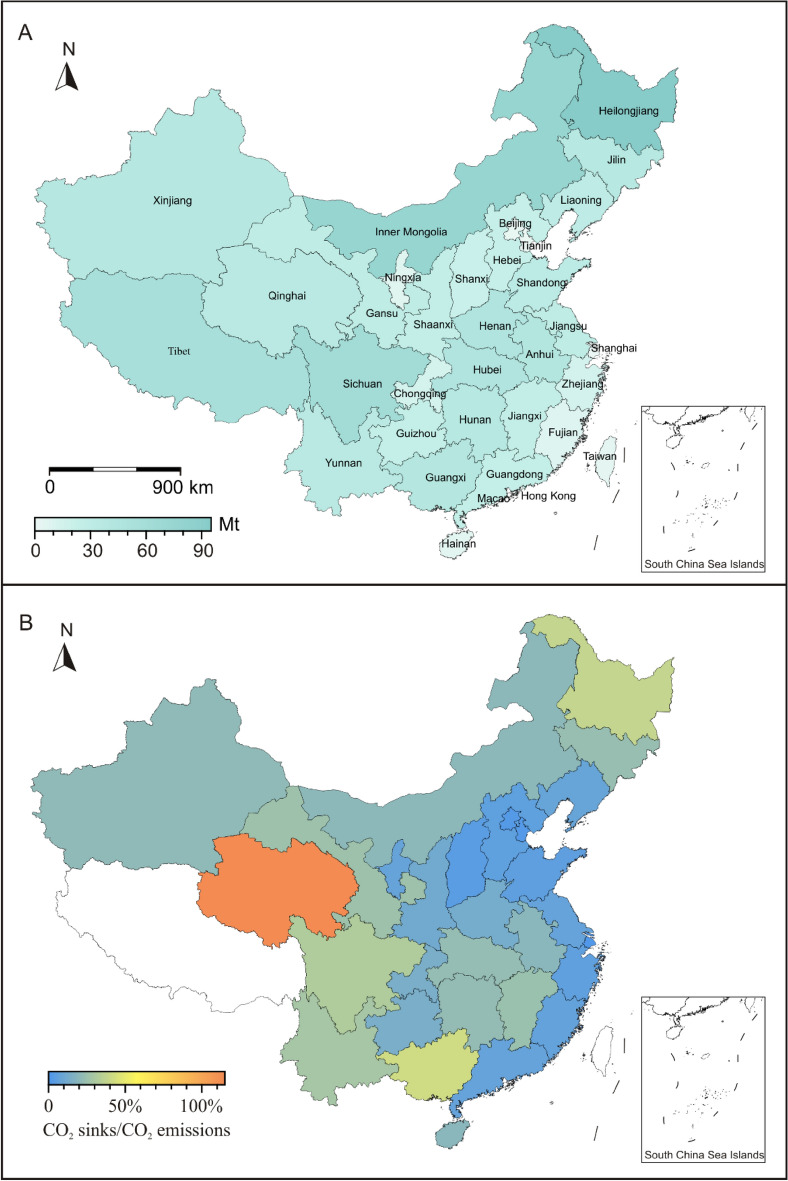
Map showing the province-level terrestrial ecosystem CO_2_ uptakes and the ratio of CO_2_ uptakes to CO_2_ emissions. (**A**) The annual average of provinces-level terrestrial ecosystem CO_2_ uptakes for the period 1982–2015. No data in Macao. (**B**) The ratios of average CO_2_ uptakes to contemporary CO_2_ emissions for the period 1982–2015 in China. No data in Tibet, Hong Kong, Macao, and Taiwan. Map was drawn by the Generic Mapping Tools package (version 5.4.1) based on the standard map of China No. GS (2023) 2767.

Based on CEVSA model, China’s terrestrial ecosystem yields carbon sinks of ~ 266.2 Tg C/a (or CO_2_ uptakes of ~ 976.2 Mt/a) for the period 1982–2015. Overall, the northern and western China yield higher values than the rest of areas (Fig. 7A, Table 1). Among 33 provinces, Heilongjiang province has the largest CO_2_ uptakes with value of 94.7 Mt/a, while Hong Kong yields the smallest value of 0.1 Mt/a.

**Table 1:** Province-level terrestrial ecosystem carbon sinks and CO_2_ uptakes based on CEVSA model.

Province	Carbon sinks (Tg C/a)	CO_2_ uptakes (Mt/a)
Anhui	11.74	43.06
Beijing	0.42	1.55
Chongqing	3.69	13.54
Fujian	2.30	8.42
Gansu	8.18	30.00
Guangdong	6.47	23.73
Guangxi	11.25	41.26
Guizhou	6.70	24.56
Hainan	1.39	5.08
Hebei	6.36	23.32
Henan	12.42	45.54
Heilongjiang	25.83	94.72
Hong Kong	0.02	0.06
Hubei	12.33	45.19
Hunan	10.50	38.49
Inner Mongolia	20.78	76.18
Jilin	10.59	38.84
Jiangsu	8.16	29.93
Jiangxi	6.80	24.92
Liaoning	8.43	30.93
Ningxia	1.93	7.07
Qinghai	9.58	35.12
Shandong	9.03	33.11
Shaanxi	7.23	26.52
Shanxi	5.69	20.86
Shanghai	0.25	0.90
Sichuan	16.93	62.09
Taiwan	1.17	4.30
Tianjin	0.40	1.47
Tibet	15.37	56.34
Xinjiang	10.77	39.48
Yunnan	9.57	35.10
Zhejiang	3.95	14.47
Macao	No data	No data
Total	266.23	976.17

Annual carbon sequestration by China’s terrestrial ecosystems offsets 18.3% of its CO_2_ emissions for the studied period. The northern and western China have higher ratios of CO_2_ uptakes compared to CO_2_ emissions (mainly ranging from 20 to 40%) than the other provinces (< 20%, Fig. 7B). Notably, Qinghai is the only province with CO_2_ uptakes higher than coeval CO_2_ emissions.

## Discussion

Four groups’ growth patterns of energy consumptions and CO_2_ emissions result from the integration of a plethora of socioeconomic factors. In detail, provinces from the first group yield increasing energy consumption and CO_2_ emissions for the period 1997–2019. However, the increment rate of CO_2_ emissions is slow compared to that of energy consumption since 2013 (Figs. 3A, 4A). This is attributed to the energy transformation around 2013, i.e., the decreasing percentage of coal in total energy consumption. For example, raw coal consumed in Fujian province decreased from 2.63 × 10^7^ tonnes in 2015 to 2.24 × 10^7^ tonnes in 2019, while the value of natural gas increased from 3.2 × 10^9^ m^3^ in 2015 to 3.8 × 10^9^ m^3^ in 2019, respectively^6^. Correspondingly, city-level energy intensity in Fujian province generally shows a stable or decreasing trend since 2013 based on STIRPAT-PLSR model^41^.

**Figure 8:**
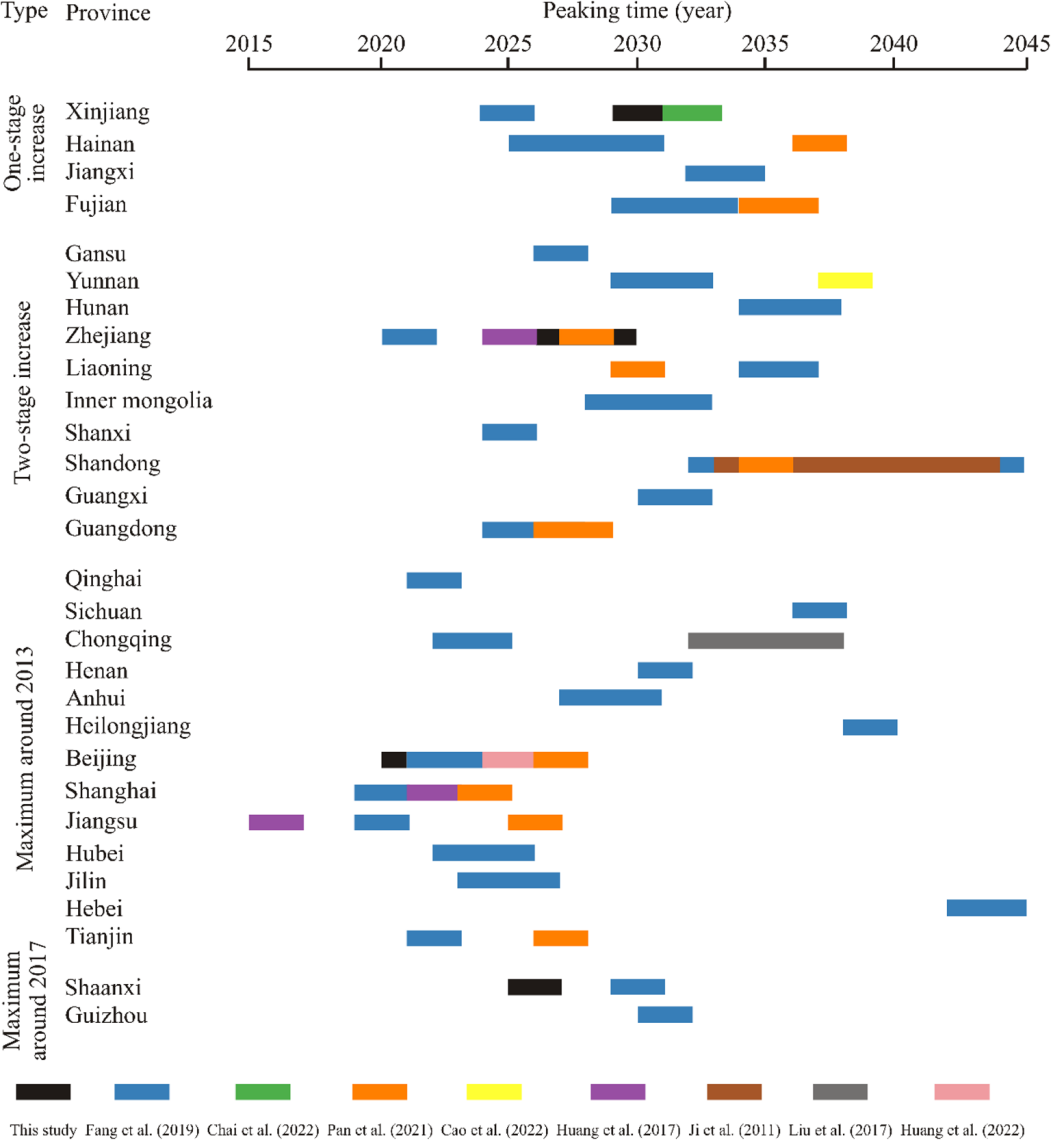
The anticipated time of CO_2_ peak emissions of 29 provinces from four groups based on STIRPAT and Markov models from this study, and previous studies^17,31,43,46–48,61,62^.

Xinjiang Uygur autonomous region from the first group shows higher carbon emission intensity (4.0) compared to Zhejiang (0.8), Beijing (0.5) and Shaanxi provinces (0.2) from other groups. Moreover, the fossil fuels consumed by Xinjiang Uygur autonomous region are dominated by coal, which accounts for 68.9% in the total energy consumption in 2020. Coal has higher carbon emission default values than oil and gas^42^. These factors likely result in the slow pathway and high emissions towards the peak carbon emissions around 2025–2030 based on ridge regression (Fig. 6A). STIRPAT models in previous studies^31,43,44^ also indicate that the optimal years of carbon emissions peak for “one-stage increase” group are around 2025–2035 (Fig. 8). Notably, the annual increment rates of factors in these studies are lower than the 14th Five-Year Plan.

The second group is characterized by an increasing energy consumption and CO_2_ emissions until 2012 or 2013, and then shows significant decrease in the next year, and finally yields stable increase in the following years (Figs. 3B, 4B). Notably, Shanxi and Inner Mongolia yield the highest and second highest increment rate of CO_2_ emissions from 2002 to 2013 due to the high volume of coal consumption (Fig. 2). These two provinces are the primary coal producers and consumers in China, and the percentage of produced coal in the national context is 21.1% for Shanxi and 20.7% for Inner Mongolia in 2009^4^, respectively. Shanxi hosts the China National Coal Group Corporation, and Inner Mongolia is the base of the China Shenhua Energy Company Limited. These two enterprises are the only two Chinese central State-owned enterprises focusing on coal.

Zhejiang province from the second group has higher urbanization rate (71.6%) than Xinjiang Uygur autonomous region (51.5%) and Shaanxi province (59.4%) in 2019, while the exponential parameter of urbanization rate shows an increasing trend from Zhejiang (0.278) to Xinjiang (0.451) and Shaanxi province (0.921). This could be explained by the positive effect of urbanization on carbon emission^45^. Although Zhejiang province has larger population than Xinjiang Uygur autonomous region and Shaanxi province, the significantly small percentage of coal consumption in total energy consumption leads to the relatively low peak emissions (0.387 billion tonnes) around 2025–2030 based on ridge regression (Fig. 6B). Likewise, 0.385–0.393 billion tonnes during 2023–2028 are proposed based on ordinary least square regression^32^. STIRPAT and Markov models suggest that this group generally reaches peak emissions during the period 2025–2035^17,43,46–48^ (Fig. 8).

The third group is characterized by the peak values of historic energy consumption and CO_2_ emissions occurring in 2012 or 2013, with subsequent decrease or stability in the following years (Figs. 3C, 4C). This is due to that these provinces followed national policies announced in 2013 about energy transition and the expand of the terrestrial ecosystem^49^. Compared to Xinjiang, Zhejiang and Shaanxi provinces, Beijing from the third group has lower carbon emissions intensity, coal consumption, energy consumption intensity, and industrial structure values. In detail, the fossil fuels consumed in Beijing are dominated by oil and gas, and the percentage of coal in energy consumption is suggested to be 0.9% in 2025 according to the 14th Five-Year Plan. Moreover, geothermal and solar energy show an increasing percentage in energy consumption. These factors favourably contribute to the early anticipated time (2020–2025) of peak emissions (Fig. 6C). Compared to previous studies^17,50^, more technical factors are considered in this model. Moreover, STIRPAT and Markov models suggest that the third group preferentially reaches carbon emissions peak and carbon neutrality compared to other groups (Fig. 8). Previous studies^51^ in the European countries also show a positive impact of renewable energy consumption on the decrease of CO_2_ emissions.

The last group yields similar CO_2_ emissions variation to the “one-stage increase” group before 2017, but then shows a significant decrease (Fig. 4D). This is likely related to regional government policies to promote the adjustment of energy systems, economic development and industrial structures around 2017. For example, high-emitting coal consumed by Guizhou province is 5.25 × 10^7^, 4.3 × 10^7^ and 4.25 × 10^7^ tonnes in 2017, 2018 and 2019, respectively^40^. Clean gas increased from 1.4 × 10^9^ m^3^ in 2017 to 3.2 × 10^9^ m^3^ in 2019. Likewise, energy transformation and economic growth have significant effects on carbon emissions of Shaanxi province^52^. STIRPAT model shows that Shaanxi province likely reaches peak emissions around 2025 only in the context of high-speed scenario with significant decrease of the percentage of coal in energy consumption. The current percentage is 75.3%, likely contributing to the high carbon emissions. Likewise, this province is suggested to achieve peak emissions around 2030 in low carbon emission intensity, and no peak values in other scenarios^15^. The slight difference compared to this study might be caused by the anticipated increment rates of GDP per capita and energy consumption intensity.

In summary, the first group (e.g., Xinjiang) and second group (e.g., Zhejiang province) might reach carbon emission peak before 2030. The third group (e.g., Beijing) likely achieves peak emission around 2020–2025 (Fig. 8). However, the fourth group (e.g., Shaanxi province) needs to take strict measures to reach peak emissions before 2030. Although three types of scenarios with several factors are incorporated into STIRPAT model, this study acknowledges that there could be additional uncertainties for these large provincial emitters^9^.

From the point of carbon sinks, China’s terrestrial ecosystem yields carbon sinks of ~ 266.2 Tg C/a (CO_2_ uptakes of ~ 976.2 Mt/a) over the period 1982–2015 based on CEVSA model (Fig. 7, Table 1). This agrees with previous estimates for the same ecosystem based on ground observations with sinks of 176.9 Tg C/a over the period 1981–2000^20^. A nested atmospheric inversion system shows that terrestrial carbon sinks in China are 280 ± 180 Tg C/a for the period 2002–2008^23^. Moreover, previous work^25^ synthesizes these published data, and estimates terrestrial carbon sinks of 200–250 Tg C/a in China during the past decades. Using data calculated in this study, carbon sequestration by China’s terrestrial ecosystems offsets 18.3% of contemporary CO_2_ emissions. Likewise, the proportion of China’s CO_2_ emission offset by contemporary terrestrial carbon sink gradually decreases from ~ 30% in the 1980s to 7–15% since 2010^22^. The authors acknowledge that the values of terrestrial carbon sinks calculated in this study and previous studies show some uncertainties and differences. These are likely caused by observations parameters, model structures and other factors. Integrating new observations and improving algorithms have potential to meet these challenges.

Provinces in the northern and western China, yield higher terrestrial carbon sinks than other provinces in China. The terrestrial ecosystems in the northern and western provinces generally offset ~ 20–40% of their CO_2_ emissions, while other provinces yield low values (< 20%). This might be related to the better ecological systems, larger provincial area and well-preserved grassland and forest in the northern and western China. For example, Heilongjiang province, located in the northern China, yields the highest CO_2_ uptakes (94.7 Mt/a), and could offset 38% of contemporary CO_2_ emissions. This is due to that this province has a total forest area of over 10.0 million ha, accounting for ~ 31% of the total forest area in China^53^. Qinghai province in the western China is the only one with terrestrial ecosystems completely offsetting carbon emissions^54^ (Fig. 7B). Qinghai province is also suggested to have the highest level of ecological security and occur as carbon compensation in 24 provincial-level regions^24^.

## Conclusion and policy recommendations

In this paper, the growth patterns of 30 provinces’ energy consumption and CO_2_ emissions for the period 1997–2019 are categorized into four groups: (i) one-stage increase; (ii) two-stage increase; (iii) maximum around 2013, and (iv) maximum around 2017. Both Xinjiang Uygur autonomous region from the first group and Zhejiang province from the second group likely achieve CO_2_ emissions peak during 2025–2030. Beijing from the third group could preferentially reach peak carbon emissions during 2020–2025. Shaanxi province from the fourth group is difficult to reach peak values prior to 2030. Compared to other groups, Beijing is characterized by low carbon emissions intensity and increasing renewable energy consumption, favourable for reaching carbon emissions peak and carbon neutrality. China’s terrestrial ecosystem over the period 1982–2015 yields carbon sinks of ~ 266.2 Tg C/a, offsetting 18.3% of contemporary carbon emissions. The ecological systems and well-preserved grassland and forest likely lead to high terrestrial carbon sinks in the northern and western China.

Based on the above research findings, policy suggestions given here are as follows: (i) the clean transformation of energy systems. The energy mix is projected to shift from high-carbon coal and oil towards low-carbon solutions, with particularly important players for solar, wind, nuclear, hydro and geothermal^55,56^. Deep decarbonization entails low-emitting and high-efficiency technologies when consuming these energies. Nuclear might supply a more consistent base load of power than solar and wind. These are crucial for the first, second and fourth groups to achieve CO_2_ emissions peak before 2030. (ii) green and digital technology^57–59^, e.g., carbon capture and storage (CCS). Carbon capture and storage technology could capture CO_2_ released from burning fossil fuels or biomass, and store it underground. Approximately 850 gigawatts of power generated from fossil fuels are estimated to be fitted with this technology in China by 2060^60^. Moreover, carbon dioxide flooding technology is performed to store carbon emissions and flood oil in many provinces, China. (iii) terrestrial ecosystem. The terrestrial ecosystem is efficient in the uptake of atmosphere carbon dioxide and slowness of climate warming. Science-based ecological engineering measures (e.g., reforestation, suitable grazing, and diversity-carbon co-benefit) are urgent to be performed to maximize its contribution to the ‘carbon neutrality’ strategy.

### Supplementary Information

Supplementary Information.

## Data Availability

The datasets generated during the current study are available from the corresponding author on reasonable request.
