# Supplementary material for: The trajectory of carbon emissions and terrestrial carbon sinks at the provincial level in China
Source: Sci Rep. 2024 Mar 9;14:5828. doi: 10.1038/s41598-024-55868-y (PMC10925036; doi:10.1038/s41598-024-55868-y)
Supplement: Supplementary file 1 — Supplementary Information. [file 41598_2024_55868_MOESM1_ESM.docx]

**The trajectories of CO_2_ emissions and terrestrial carbon sinks of 30 Chinese provinces**

Yongjie Hu^1^, Ying Li^2, *^, Hong Zhang^1^, Xiaolin Liu^3, *^, Yixian Zheng^1^ & He Gong^4^

^1^Sinopec International Petroleum Exploration and Production Corporation, Beijing 100029, China

^2^School of Earth Sciences and Engineering, Nanjing University, Nanjing, Jiangsu 210093, China

^3^Merchant Marine College, Shanghai Maritime University, Shanghai, 201306, China

^4^Key Laboratory of Ecosystem Network Observation and Modeling, Institute of Geographic Sciences and Natural Resources Research, Chinese Academy of Sciences, Beijing 100101, China

**Corresponding author: Ying Li (yingli@smail.nju.edu.cn); Xiaolin Liu (*[*xlliu2@shmtu.edu.cn*](mailto:xlliu2@shmtu.edu.cn)*)*

**Supplementary Table S1.** Scenarios (low-speed, business-as-usual (BAU) and high-speed) setup for Xinjiang, Zhejiang, Beijing and Shaanxi provinces.

| Province or region | Scenario | Period (year) | Population (P) | GDP per capita (A) | Carbon emission intensity (ET) | Energy consumption intensity (EC) | Energy structure (ES) | Industrial structure (IS) | Urbanization rate (UR) |
| --- | --- | --- | --- | --- | --- | --- | --- | --- | --- |
| Xinjiang Uygur autonomous region | Low | 2021-2025 | 0.08% | 5.80% | -4.50% | -2.70% | -3.30% | -2.50% | 0.80% |
|  |  | 2026-2030 | 0.06% | 5.60% | -4.00% | -2.40% | -3.10% | -2% | 0.60% |
|  |  | 2031-2035 | 0.04% | 5.40% | -3.50% | -2.10% | -2.90% | -1.50% | 0.40% |
|  |  | 2036-2050 | 0.02% | 5.20% | -3% | -1.80% | -2.70% | -1% | 0.20% |
|  | BAU | 2021-2025 | 1% | 6.00% | -5.00% | -3.20% | -3.80% | -3% | 1.00% |
|  |  | 2026-2030 | 0.08% | 5.80% | -4.50% | -2.90% | -3.60% | -2.50% | 0.80% |
|  |  | 2031-2035 | 0.06% | 5.60% | -4.00% | -2.60% | -3.40% | -2% | 0.60% |
|  |  | 2036-2050 | 0.04% | 5.40% | -3.50% | -2.30% | -3.20% | -1.50% | 0.40% |
|  | High | 2021-2025 | 1.20% | 6.50% | -5.50% | -3.70% | -4.30% | -3.50% | 1.20% |
|  |  | 2026-2030 | 1% | 6.30% | -5.00% | -3.40% | -4.10% | -3% | 1.00% |
|  |  | 2031-2035 | 0.08% | 6.10% | -4.50% | -3.10% | -3.90% | -2.50% | 0.80% |
|  |  | 2036-2050 | 0.06% | 5.90% | -4.00% | -2.80% | -3.70% | -2% | 0.60% |
| Zhejiang | Low | 2021-2025 | 1% | 5.50% | -4.50% | -2.90% | -5.00% | -1.80% | 0.60% |
|  |  | 2026-2030 | 0.80% | 5.00% | -4.00% | -2.60% | -4.80% | -1.60% | 0.40% |
|  |  | 2031-2035 | 0.60% | 4.50% | -3.50% | -2.30% | -4.60% | -1.40% | 0.20% |
|  |  | 2036-2050 | 0.40% | 4.00% | -3% | -2% | -4.40% | -1.20% | 0.10% |
|  | BAU | 2021-2025 | 1.20% | 6% | -5.00% | -3.20% | -5.00% | -2% | 1% |
|  |  | 2026-2030 | 1% | 5.50% | -4.50% | -3.00% | -4.80% | -1.80% | 0.80% |
|  |  | 2031-2035 | 0.80% | 5.00% | -4.00% | -2.80% | -4.60% | -1.60% | 0.60% |
|  |  | 2036-2050 | 0.60% | 4.50% | -3.50% | -2.60% | -4.40% | -1.40% | 0.40% |
|  | High | 2021-2025 | 1.40% | 6.50% | -6.00% | -3.50% | -5.40% | -2.20% | 1.20% |
|  |  | 2026-2030 | 1.20% | 6% | -5.50% | -3.20% | -5.20% | -2% | 1% |
|  |  | 2031-2035 | 1% | 5.50% | -5.00% | -2.90% | -5.00% | -1.80% | 0.80% |
|  |  | 2036-2050 | 0.80% | 5.00% | -4.50% | -2.60% | -4.80% | -1.60% | 0.60% |
| Beijing | Low | 2021-2025 | 0.08% | 6.50% | -4.50% | -2.90% | -4.00% | -3.50% | 0.05% |
|  |  | 2026-2030 | 0.06% | 5.50% | -4% | -2.60% | -3.80% | -3% | 0.05% |
|  |  | 2031-2035 | 0.04% | 4.50% | -3.50% | -2.30% | -3.60% | -2.50% | 0.05% |
|  |  | 2036-2050 | 0.02% | 4.00% | -3.00% | -2% | -3.40% | -2% | 0.05% |
|  | BAU | 2021-2025 | 1% | 7% | -5.00% | -3.20% | -5.00% | -4% | 0.10% |
|  |  | 2026-2030 | 0.08% | 6.00% | -4.50% | -2.90% | -4.80% | -3.50% | 0.10% |
|  |  | 2031-2035 | 0.06% | 5.00% | -4.00% | -2.60% | -4.60% | -3% | 0.10% |
|  |  | 2036-2050 | 0.04% | 4.50% | -3.50% | -2.30% | -4.40% | -2.50% | 0.10% |
|  | High | 2021-2025 | 1.20% | 7.20% | -6.50% | -3.80% | -6.00% | -5.00% | 0.17% |
|  |  | 2026-2030 | 1% | 6.20% | -6.00% | -3.50% | -5.80% | -4.50% | 0.15% |
|  |  | 2031-2035 | 0.08% | 5.20% | -5.50% | -3.20% | -5.60% | -4.00% | 0.13% |
|  |  | 2036-2050 | 0.06% | 4.70% | -4.50% | -2.90% | -5.40% | -3.50% | 0.11% |
| Shaanxi | Low | 2021-2025 | 0.08% | 5.50% | -4.50% | -2.70% | -1.20% | -2.50% | 0.80% |
|  |  | 2026-2030 | 0.06% | 5.30% | -4.00% | -2.40% | -1.00% | -2% | 0.60% |
|  |  | 2031-2035 | 0.04% | 5.10% | -3.50% | -2.10% | -0.80% | -1.50% | 0.40% |
|  |  | 2036-2050 | 0.02% | 4.90% | -3% | -1.80% | -0.60% | -1% | 0.20% |
|  | BAU | 2021-2025 | 1% | 6.00% | -5.00% | -3.20% | -1.50% | -3% | 1.50% |
|  |  | 2026-2030 | 0.08% | 5.80% | -4.50% | -2.90% | -1.30% | -2.50% | 1.30% |
|  |  | 2031-2035 | 0.06% | 5.60% | -4.00% | -2.60% | -1.10% | -2% | 1.10% |
|  |  | 2036-2050 | 0.04% | 5.40% | -3.50% | -2.30% | -0.90% | -1.50% | 0.90% |
|  | High | 2021-2025 | 1.20% | 6.50% | -5.50% | -3.70% | -1.60% | -3.50% | 1.70% |
|  |  | 2026-2030 | 1% | 6.30% | -5.00% | -3.40% | -1.60% | -3.30% | 1.50% |
|  |  | 2031-2035 | 0.08% | 6.10% | -4.50% | -3.10% | -1.50% | -3.10% | 1.30% |
|  |  | 2036-2050 | 0.06% | 5.90% | -4.00% | -2.80% | -1.50% | -2.90% | 1.10% |
